# Analysis of 92 hospitalized cases of non-neonatal tetanus in Guizhou Province, China

**DOI:** 10.3389/fpubh.2026.1951130

**Published:** 2026-09-30

**Authors:** Jiao Li, Dandan Zhang, Ruizhi Zhang, Leibing Zhang, Bin Qu, Yan Zhang, Luyu Tang, Jun Li, Ming Liu

**Affiliations:** 1 School of Public Health, Key Laboratory of Environmental Pollution Monitoring and Disease Control, Ministry of Education, Guizhou Medical University, Guiyang, China; 2 Guizhou Provincial Center for Disease Control and Prevention, Department of Immunization Program, Guiyang, Guizhou, China; 3 Guizhou Provincial People's Hospital, Department of Emergency Surgery, Guiyang, Guizhou, China; 4 Zunyi Center for Disease Control and Prevention, Zunyi, Guizhou, China; 5 Nanming District Center for Disease Control and Prevention, Guiyang, Guizhou, China

**Keywords:** characteristics of cases, disease burden, hospitalization expenses, non-neonatal, tetanus

## Abstract

**Background:**

Tetanus has a high mortality rate and remains a major public health threat in low- and middle-income countries. Although neonatal tetanus has been eliminated in China, the burden of non-neonatal tetanus is still heavy. This study analyzed the case characteristics, outcomes, and hospitalization expenses of non-neonatal tetanus inpatients in Guizhou Province, China, and provided a basis for reducing the disease burden.

**Methods:**

One tertiary medical institution was randomly selected from each of the nine cities (states) in the province, and the data of non-neonatal tetanus hospitalized cases from 2021 to 2025 were collected. Descriptive epidemiological methods, the simple seasonal index method, and multiple linear regression were used in the analysis.

**Results:**

A total of 92 cases were included. Males accounted for 65.22% (60/92); farmers accounted for 86.96% (80/92); the median age was 60 (55–69) years; the incubation period ranged from 1 to 27 days; and cases peaked in summer. 95.65% (88/92) were not vaccinated with tetanus toxoid-containing vaccine (TTCV), and only 21.74% (20/92) received standardized treatment within 24 h after injury. The case fatality rate was 15.22% (14/92). None of the non-survivors were inoculated with TTCV, no standardized treatment was performed within 24 h after injury, the severity was severe or above, and the family members gave up treatment on their own. The median hospitalization cost was 55,724 yuan (equivalent to 8,359 US Dollars); the highest was 239,365 yuan (equivalent to 35,905 US Dollars); and the number of hospitalization days was the main influencing factor (*t* = 5.751, *p* < 0.001).

**Conclusion:**

Non-neonatal tetanus in Guizhou Province was mainly in middle-aged and older male farmers, with a high incidence in summer, and most of them were not vaccinated with TTCV. The non-survivors were not vaccinated with TTCV and did not receive standard treatment within 24 h after injury. The disease had a high case fatality rate and was a serious economic burden. It is recommended to strengthen TTCV vaccination and standardized post-exposure disposal in high-risk groups, enhance medical financial support to prevent abandonment of treatment for economic reasons, and ensure that patients receive timely and comprehensive clinical care.

## 1 Introduction

Tetanus is a serious and potentially fatal acute infectious disease caused by *Clostridium tetani*. Its typical clinical manifestations are generalized skeletal muscle tonic contraction and paroxysmal spasm (1). The case fatality rate of tetanus was close to 100% without any treatment. Under the condition of active comprehensive treatment, 10,000 to 300,000 patients worldwide still die of tetanus every year, and the case fatality rate is between 5 and 48% (2, 3).

China successfully eliminated maternal and neonatal tetanus in 2012 after introducing the DPT vaccine into its immunization program in 1978 (4). However, non-neonatal tetanus remains an important public health challenge. According to the World Health Organization and the United Nations International Children’s Emergency Fund, 85% of global tetanus cases in 2016 were non-neonatal tetanus (5). Studies have shown that the per capita hospitalization cost for tetanus patients is as high as 83,097 yuan, about 12.93 times China’s per capita annual total health expenditure, which imposes a serious economic burden on patients, their families, and society (6).

At present, non-neonatal tetanus is not included in China’s statutory infectious disease reporting system, and its epidemiological and clinical data are scarce. Guizhou Province is located in southwestern China and has a relatively weak economic foundation. Systematic research on non-neonatal tetanus in this area is extremely limited. Therefore, this study collected data on non-neonatal tetanus inpatients at several tertiary hospitals in Guizhou Province from 2021 to 2025, aiming to systematically analyze their clinical characteristics and the factors influencing hospitalization expenses, and to provide a basis for reducing the burden of tetanus in this area.

## 2 Materials and methods

### 2.1 Study design and case inclusion

This study was approved by the Ethics Committee of the Guizhou Provincial Center for Disease Control and Prevention (Approval No.: W2023-10), and written informed consent was obtained from all participants or their immediate family members. The study employed a retrospective survey method, randomly selecting one tertiary medical institution from each of the nine cities (prefectures) in Guizhou Province, and retrieving inpatient cases diagnosed with ‘tetanus’ from the hospital management system, with admission dates between January 1, 2021, and December 31, 2025.

Inclusion criteria: (1) clinically confirmed diagnosis of ‘tetanus’; (2) Meeting the diagnostic criteria in the ‘Guidelines for the Diagnosis and Treatment of Non-neonatal Tetanus (2024 Edition)’ (7): at least one of the typical clinical manifestations, including trismus, risus sardonicus, or painful muscle spasms, after excluding other etiologies. Exclusion criteria: (1) Cases of spasms or clinical manifestations similar to tetanus caused by other neurological diseases; (2) Cases with incomplete clinical data that cannot be supplemented. The severity grading of non-neonatal tetanus is classified into four levels: mild, moderate, severe, and very severe, based on the maximum distance between upper and lower incisor edges, muscle spasms, dysphagia, respiratory distress, and the degree of autonomic dysfunction (Table 1).

**Table 1:** Severity classification of non-neonatal tetanus.

Severity	Trismus (Maximum distance between upper and lower incisor edges)	Muscle spasm episodes*	Dysphagia	Respiratory distress	Autonomic dysfunction
Mild	>1 cm	None	None or mild	None	None
Moderate	1–2 cm	Mild to moderate, transient	Moderate	Respiratory rate 30–40 breaths/min	None
Severe	<1 cm	Severe, persistent	Severe	Respiratory rate >40 breaths/min, speech impairment	Heart rate >120 bpm
Very severe	<1 cm	Severe, persistent	Severe	Respiratory rate >40 breaths/min, speech impairment	Severe and persistent hypertension, tachycardia, or hypotension, bradycardia

Note: The severity grade was determined by the most severe of the patient’s indicators. *The patients were kept in a state of muscle relaxation, rapid passive activity in the whole range of joint activity. They felt the resistance caused by muscle tension during the activity. Normal: no obvious resistance; mild: felt slight muscle contraction, but had little effect on joint activity; moderate: felt obvious muscle contraction, but joint activity is still easy; severe: Joint activity is difficult or unable to perform joint activities due to muscle contraction.

### 2.2 Data collection and quality control

The case data were extracted using the ‘non-neonatal tetanus inpatient case information collection table ‘designed by the research group. The main contents include: (1) sociodemographic characteristics (gender, age, occupation); (2) exposure and injury factors (trauma history, injury site, wound type, injury-causing agents); (3) immunization history of TTCV; (4) Clinical characteristics (incubation period, length of hospital stay, severity of disease, complications, etc.); (5) Treatment (wound treatment within 24 h after injury, type of passive immunization, ICU admission, mechanical ventilation, tracheotomy); (6) Hospitalization expenses and outcome.

All case data were verified with medical staff, the patient, and their family members to supplement any missing information. For cases in which family members voluntarily withdrew from treatment, their outcomes (survival or non-survival) were confirmed via telephone follow-up. Two researchers independently entered and cross-checked the case data, and those with inconsistent entries were asked to check the original survey data to verify the correction. Based on the case outcomes, cases were divided into survival and non-survival groups for comparative analysis.

### 2.3 Hospitalization expenses calculation

Hospitalization expenses were the direct medical costs incurred by tetanus patients during their hospital stays. Data were extracted from each hospital’s medical record management system, including comprehensive medical service fees, diagnostic fees, treatment fees, drug fees (including traditional Chinese and Western medicines), and other related expenses (such as rehabilitation, blood products, and consumables). All expenses were based on the patient discharge settlement amount, and the unit is RMB (yuan). To facilitate international readers’ understanding, this study provides the exchange rate conversion: 1 yuan is approximately $0.15, and readers can convert the costs in the text to the equivalent dollar amount using this ratio.

### 2.4 Statistical analysis

Excel 2010 software was used for data collation and drawing, and Jamovi 2.6.17.0 software was used for data analysis. Measurement data with non-normal distribution were expressed as median (quartile) [*M* (*P_25_*-*P_75_*)], and the categorical data were expressed as rate or constituent ratio (%). The simple seasonal index method was used to describe the time distribution of cases. The simple seasonal index is calculated as the ratio of the average number of cases for a specific month across all years to the average number of cases across all months and all years. A simple seasonal index ≥1 indicates seasonality; otherwise, it does not. Spearman’s rank correlation was used to analyze the correlation of hospitalization expenses. Mann–Whitney U or Kruskal-Wallis H rank sum test was used for non-normally distributed continuous data, and chi-square test or Fisher’s exact test was used for categorical data. For multivariate analysis, multiple linear regression was used to assess factors influencing hospitalization expenses. Because hospitalization expenses were not normally distributed, they were included in the multiple linear regression model after being logarithmically transformed. A two-sided *p*-value <0.05 was considered statistically significant.

## 3 Results

### 3.1 The basic situation of the 92 cases

We initially screened the inpatient medical record systems of nine tertiary hospitals and identified 97 suspected cases diagnosed as “tetanus.” After excluding 5 cases based on strict review of clinical data against the inclusion and exclusion criteria, we included 92 non-neonatal tetanus cases. Among them, men accounted for 65.22% (60/92). The age ranged from 27 to 85 years, with a median of 60 (55–69) years. The incubation period ranged from 1 to 27 days, with a median of 10 (7–13.25) days. Length of hospitalization ranged from 1 to 57 days, with a median of 17 (7–20) days. Farmers were the main occupation, accounting for 86.96% (80/92). Case counts exhibited a seasonal pattern, with most cases occurring in summer. The seasonal indices in June, July, and August were 2.09, 2.09, and 1.04, respectively (Table 2; Figure 1).

**Table 2:** Clinical data of 92 cases of non-neonatal tetanus.

Variables	Non-Survivors [n (%)/*M*(*P*_25_-*P*_75_)]	Survivors [n (%)/*M*(*P_25_*-*P_75_*)]	*Z*/ *χ^2^*	*p*	Total cases [n (%)/*M* (*P_25_*-*P_75_*)]
Age (years)	63.50 (58.75–74.75)	59.00 (55.00–68.00)	432.500	0.219	60.00 (55.00–69.00)
Incubation period (days)	7.00 (6.00–7.00)	11.00 (8.00–14.00)	189.500	<0.001	10.00 (7.00–13.25)
Length of hospitalization (days)	15.00 (11.25–23.00)	17.00 (7.00–30.00)	476.000	0.450	17.00 (7.00–20.00)
Gender					
Male	10 (71.43)	50 (64.10)	0.281	0.596	60 (65.22)
Female	4 (28.57)	28 (35.90)			32 (34.78)
Occupation			0.079	0.779	
Farmer	13 (92.86)	67 (85.90)			80 (86.96)
Others	1 (7.14)	11 (14.10)			12 (13.04)
Injury site				0.948*	
Lower limbs	6 (46.15)	28 (38.89)			34 (40.00)
Upper limbs	5 (38.46)	26 (36.11)			31 (36.47)
Head/face	2 (15.38)	16 (22.22)			18 (21.18)
Multiple injuries	0 (0.00)	2 (2.78)			2 (2.35)
Wound type				0.982*	
Puncture wound	3 (23.08)	19 (26.39)			22 (25.88)
Contusion	2 (15.38)	15 (20.83)			17 (20.00)
Laceration	2 (15.38)	12 (16.67)			14 (16.47)
Fall-related injuries	2 (15.38)	10 (13.89)			12 (14.12)
Crush injury	2 (15.38)	6 (8.33)			8 (9.41)
Burn	1 (7.69)	6 (8.33)			7 (8.24)
Others	1 (7.69)	4 (5.56)			5 (5.88)
Injury-causing agents				0.982*	
Rusted objects	4 (30.77)	15 (20.83)			19 (22.35)
Ground surface	3 (23.08)	16 (22.22)			19 (22.35)
Stones	2 (15.38)	10 (13.89)			12 (14.12)
Knife or sharp objects	2 (15.38)	9 (12.50)			11 (12.94)
Wood	0 (0.00)	8 (11.11)			8 (9.41)
Flammable or explosive items	1 (7.69)	6 (8.33)			7 (8.24)
Others	1 (7.69)	8 (11.11)			9 (10.59)
Wound treatment within 24 hours				<0.001*	
No treatment	11 (78.57)	17 (21.79)			28 (30.43)
Self-treatment	2 (14.29)	34 (43.59)			36 (39.13)
Hospital-standard treatment	0 (0.00)	20 (25.64)			20 (21.74)
Unknown	1 (7.14)	7 (8.97)			8 (8.70)
TTCV vaccination status				1.000*	
Yes	0 (0.00)	4 (5.13)			4 (4.35)
No	14 (100.00)	74 (94.87)			88 (95.65)
Type of passive immunization				0.012*	
Human tetanus immunoglobulin	13 (92.86)	43 (55.13)			56 (60.87)
Equine tetanus immunoglobulin or tetanus antitoxin	0 (0.00)	26 (33.33)			26 (28.26)
Tetanus antitoxin and human tetanus immunoglobulin	1 (7.14)	9 (11.54)			10 (10.87)
Severity				<0.001*	
Mild	0 (0.00)	26 (33.33)			26 (28.26)
Moderate	0 (0.00)	28 (35.90)			28 (30.43)
Severe	7 (50.00)	24 (30.77)			31 (33.70)
Very severe	7 (50.00)	0 (0.00)			7 (7.61)
History of underlying diseases			0.191	0.663	
Yes	4 (28.57)	15 (16.11)			19 (20.65)
No	10 (71.43)	63 (80.77)			73 (79.35)
ICU admission			9.680	0.002	
Yes	14 (100.00)	44 (56.41)			58 (63.04)
No	0 (0.00)	34 (43.59)			34 (36.96)
Mechanical ventilation			5.291	0.021	
Yes	14 (100.00)	51 (65.38)			65 (70.65)
No	0 (0.00)	27 (34.62)			27 (29.35)
Tracheostomy			10.620	0.001	
Yes	12 (85.71)	33 (42.31)			45 (48.91)
No	2 (14.29)	45 (57.67)			47 (51.09)
Discharge outcome				<0.001*	
Cured	0 (0.00)	12 (15.38)			12 (13.04)
Improved discharge	0 (0.00)	60 (76.92)			60 (65.22)
Family abandoned treatment	14 (100.00)	6 (7.69)			20 (21.74)
Complications			1.962	0.161	
Yes	14 (100.00)	63 (80.77)			77 (83.70)
No	0 (0.00)	15 (19.23)			15 (16.30)
Sepsis			4.333	0.037	
Yes	4 (28.57)	5 (6.41)			9 (9.78)
No	10 (71.43)	73 (93.59)			83 (90.22)
Septic shock			7.414	0.006	
Yes	6 (42.86)	8 (10.26)			14 (15.22)
No	8 (57.14)	70 (89.74)			78 (84.78)
Pulmonary infection			4.965	0.026	
Yes	14 (100.00)	52 (66.67)			66 (71.74)
No	0 (0.00)	26 (33.33)			26 (28.26)
Hypoproteinemia			10.10	0.001	
Yes	11 (78.57)	26 (33.33)			37 (40.22)
No	3 (21.43)	52 (66.67)			55 (59.78)
Multiple organ dysfunction syndrome			29.53	<0.001	
Yes	9 (64.29)	4 (5.13)			13 (14.13)
No	5 (35.71)	74 (94.87)			79 (85.87)
Upper gastrointestinal hemorrhage				0.043*	
Yes	3 (21.43)	3 (3.85)			6 (6.52)
No	11 (78.57)	75 (96.15)			86 (93.48)
Respiratory failure			45.74	<0.001	
Yes	11 (78.57)	3 (3.85)			14 (15.22)
No	3 (21.43)	75 (96.15)			78 (84.78)

*Fisher’s exact test.

**Figure 1:**
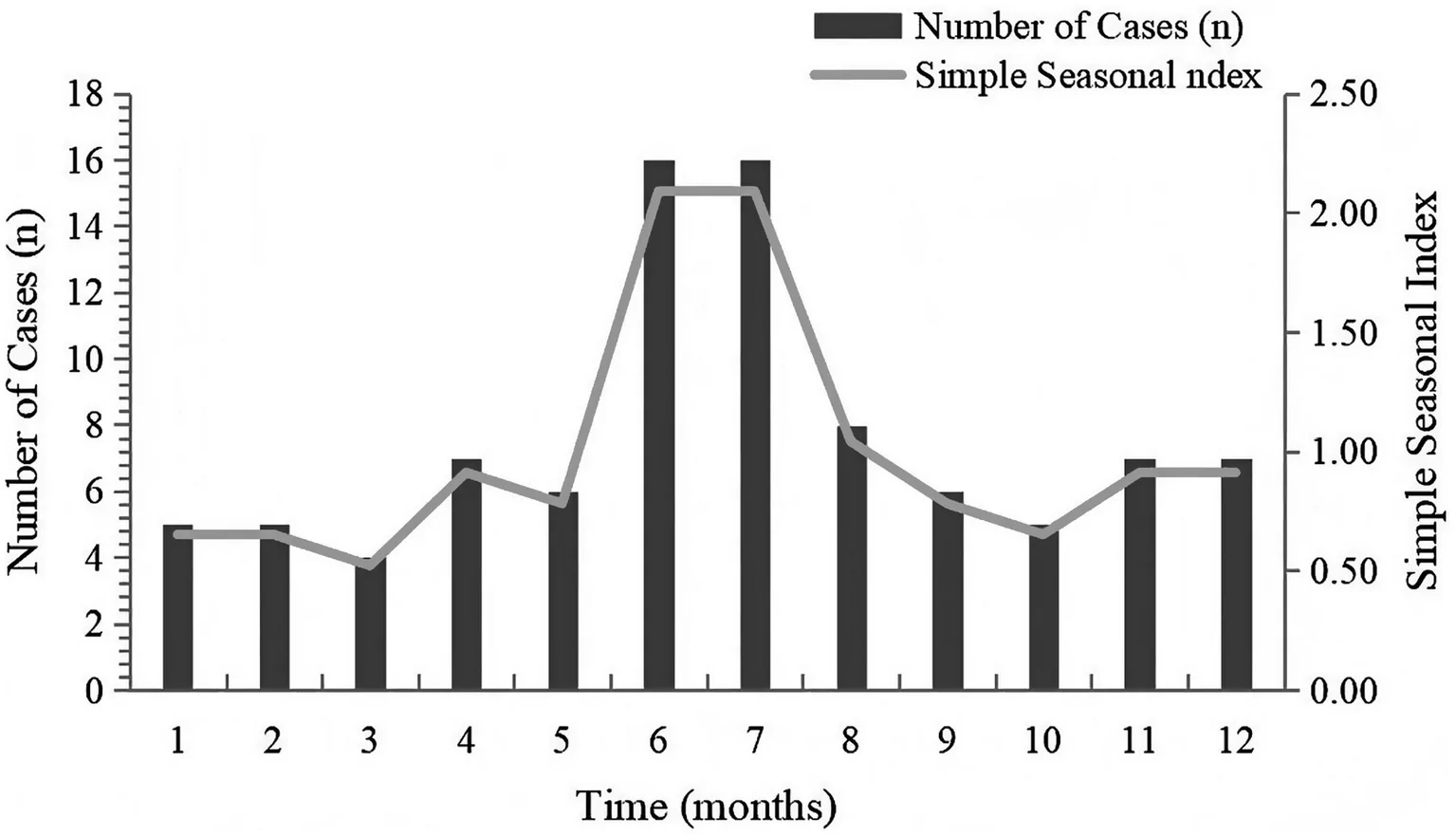
Seasonal index distribution of 92 cases of non-neonatal tetanus.

### 3.2 Injury situation and previous immunization

Of the 92 cases, 7.61% (7/92) had no clear history of trauma, while 92.39% (85/92) had a clear history of trauma. Among cases with a documented history of trauma, the most commonly affected body regions were the lower limbs (40.00%, 34/85), upper limbs (36.47%, 31/85), and head/face (21.18%, 18/85). The primary types of wounds included puncture injuries (25.88%, 22/85), contusions (20.00%, 17/85), and lacerations (21.18%, 18/85). The main injury-causing agents were rusted objects (22.35%, 19/85), ground surfaces (22.35%, 19/85), and stones (14.12%, 12/85). Only 4.35% (4/92) of cases had been vaccinated with TTCV, while 95.65% (88/92) had not (Table 2).

### 3.3 Post-injury management and clinical treatment

Only 21.74% (20/92) of cases received standardized treatment within 24 h post-injury, while 69.56% (64/92) did not. All cases received passive immunization against tetanus after admission, with 60.87% (56/92) receiving human tetanus immunoglobulin. Those admitted to the ICU accounted for 63.04% (58/92), and 70.65% (65/92) received mechanical ventilation treatment, while 48.91% (45/92) underwent tracheostomy (Table 2).

### 3.4 Clinical outcomes and influencing factors analysis

Among the 92 cases, 14 died, with a case fatality rate of 15.22% (14/92). Among them, 4 cases died in 19 cases with underlying diseases, with a case fatality rate of 21.05%; among the 73 cases without underlying diseases, 10 cases died, with a case fatality rate of 13.10%. All 14 non-survivors had not received TTCV vaccination, underwent mechanical ventilation treatment after admission, and had at least one complication, with pulmonary infection (100%, 14/14), respiratory failure (78.57%, 11/14), hypoalbuminemia (78.57%, 11/14), and multiple organ dysfunction syndrome (64.29%, 9/14) being the most common. Twelve non-survivors underwent tracheostomy, accounting for 85.72% (Table 2).

The results of univariate analysis showed that the incubation period (*Z* = 189.5, *p* < 0.001), wound treatment within 24 h (*p* < 0.001), severity (*p* < 0.001), ICU admission (*χ^2^* = 9.680, *p* = 0.002), discharge outcome (*p* < 0.001), and complications (sepsis (*χ^2^* = 4.333, *p* = 0.037), septic shock (*χ^2^* = 7.414, *p* = 0.006), pulmonary infection (*χ^2^* = 4.965, *p* = 0.026), hypoproteinemia (*χ^2^* = 10.10, *p* = 0.001), multiple organ dysfunction syndrome (*χ^2^* = 29.530, *p* < 0.001), upper gastrointestinal bleeding (*p* = 0.043), and respiratory failure (*χ^2^* = 45.74, *p* < 0.001) were statistically significant between the non-survival group and the survival group. The median incubation period in the non-survivors group (7 days) was shorter than that in the survival group (11 days). Severity grading indicated that patients in the non-survivors group had more severe conditions compared to those in the survival group, with all cases classified as severe or higher (Table 2).

### 3.5 Analysis of hospitalization expenses and influencing factors

The median hospitalization cost of 92 tetanus cases was 55,724 (14,160-149,543) yuan, with a maximum of 239,365 yuan, equivalent to approximately 8,359 (2,124-22,431) US Dollars and 35,905 US Dollars, respectively, based on an exchange rate of 6.72 RMB per USD. Correlation analysis showed that hospitalization expenses were positively correlated with hospitalization days (*r* = 0.844, *p* < 0.001) and negatively correlated with incubation period (*r* = −0.576, *p* < 0.001). The results of single-factor analysis showed that wound treatment within 24 h (*H* = 16.51; *p* < 0.001), TTCV vaccination status (*Z* = 33, *p* = 0.006), severity (*H* = 51.15; *p* < 0.001), history of underlying diseases (*Z* = 451, *p* = 0.02), ICU admission (*Z* = 286, *p* < 0.001), complications (*Z* = 166, *p* < 0.001), mechanical ventilation (*Z* = 191, *p* < 0.001), and tracheotomy (*Z* = 286, *p* < 0.001) had significant effects on hospitalization expenses. The variables statistically significant in the univariate analysis were included in the multivariate linear regression model. The results showed that hospitalization days (t = 5.751, *p* < 0.001) and history of underlying diseases (t = −2.230, *p* = 0.029) were factors influencing hospitalization expenses (Tables 3, 4).

**Table 3:** Univariate analysis of hospitalization costs in 92 cases of non-neonatal tetanus.

Variables	Hospitalization cost [*M* (*P_25_*-*P_75_*) (yuan)]	Mann–Whitney U test/ Kruskal-Wallis H test	*p*
Wound treatment within 24 hours		16.510	<0.001
No treatment	121,820 (58,821-196,097)		
Self-treatment	54,085 (14,432-105,062)		
Hospital-standard treatment	13,903 (7,599-42,153)		
Unknown	84,891 (17,946-118,141)		
TTCV vaccination status		33.000*	0.006
Yes	8,276 (6,712-9,159)		
No	61,596 (17,119-158,441)		
Severity		51.150	<0.001
Mild	11,193 (6,724-18,370)		
Moderate	58,244 (22,901-100,432)		
Severe	179,941 (90,481–206,866)		
Very severe	41,405 (14,636-96,726)		
History of underlying diseases		451.000*	0.020
Yes	18,062 (11,118-55,631)		
No	72,452 (19,061-169,020)		
ICU admission		311.000*	<0.001
Yes	95,537 (49,675 ~ 183,803)		
No	13,773 (7,599-49,675)		
Mechanical ventilation		191.000*	<0.001
Yes	92,069 (42,503-185,091)		
No	9,779 (6,850-22,258)		
Tracheostomy		286.000*	<0.001
Yes	98,993 (54,736-185,091)		
No	13,773 (7,599-23,476)		
Complications		166.000*	<0.001
Yes	75,166 (22,902-175,863)		
No	9,048 (6,850-16,138)		

*Mann–Whitney U test; other variables: Kruskal-Wallis H test.

**Table 4:** Multiple linear regression analysis of hospitalization costs in 92 cases of non-neonatal tetanus.

Variables	Regression Coefficient	*95% CI* for Regression Coefficient		*t*	*p*
Length of hospitalization (days)	0.021	0.014	0.029	5.751	<0.001
Incubation period (days)	−0.012	−0.032	0.008	−1.192	0.238
Wound Treatment Within 24 Hours (using “No treatment” as the control group)					
Unknown	0.403	−0.085	0.890	1.648	0.104
Hospital-standard treatment	−0.070	−0.313	0.173	−0.576	0.566
Self-treatment	−0.004	−0.192	0.184	−0.042	0.967
TTCV Vaccination Status	−0.077	−0.410	0.256	−0.462	0.645
Severity (using “Severe” as the control group)					
Moderate	0.142	−0.112	0.395	1.116	0.268
Very severe	−0.043	−0.361	0.275	−0.270	0.788
Mild	0.089	−0.320	0.499	0.436	0.664
History of underlying diseases	−0.193	−0.366	0.020	−2.230	0.029
ICU admission	−0.010	−0.577	0.557	−0.034	0.973
Mechanical Ventilation	−0.180	−0.583	0.223	−0.889	0.377
Tracheostomy	−0.216	−0.719	0.286	−0.858	0.394
Complications	0.208	−0.022	0.437	1.804	0.076

## 4 Discussion

Tetanus is a serious infectious disease with critical conditions and a high risk of death. It has a high incidence in developing countries, economically poor areas, and natural disaster-prone areas. Since implementing the DPT vaccine program in 1978, China has successfully eliminated neonatal tetanus (4, 8). The survey showed that the hospitalized cases of non-neonatal tetanus in Guizhou Province were mainly older male farmers, and the number of cases peaked in summer. These distribution characteristics are consistent with results from many studies at home and abroad (1, 9). *Clostridium tetani* invades the human body mainly through damaged skin or mucous membranes (10). Trauma is the most common route of infection. Guizhou Province is located in southwestern mountainous China. The outdoor environment is hot and humid in summer. Male farmers are prone to trauma due to frequent outdoor activities, thin clothing, and weak protection awareness, resulting in an increased risk of *Clostridium tetani* exposure (11). At the same time, the body’s immune function gradually declines with age, and the risk of disease after infection increases (12). It is suggested that middle-aged and older farmers in rural areas should be the key target population for primary prevention of tetanus. In this study, 92.39% of the cases had a clear history of trauma, primarily in the lower limbs, with the injuring objects mainly being rusty items and the ground, which is highly consistent with the ecological distribution characteristics of *Clostridium tetani*, which is widely present in soil and rust-contaminated dust (10). In addition, 7.61% of the hospitalized cases in this study had no clear history of trauma. The reason may be that the wound surface is too small, the superficial injury is difficult to detect, or the wound has healed before the typical symptoms appear, thereby delaying diagnosis and treatment. It is suggested that for cases with clinical manifestations consistent with the characteristics of tetanus and unclear history of trauma, it is still necessary to combine the epidemiological characteristics and clinical manifestations of tetanus to improve vigilance and avoid missed diagnosis and misdiagnosis (13, 14).

Trauma exposure alone is not sufficient to cause tetanus, and host immune status is the key factor in determining incidence. In this group of cases, 95.65% had no history of TTCV immunization, and none of the non-survivors had been vaccinated with TTCV, indicating that the lack of active immunity was the root cause of the disease. The protective efficacy of TTCV is nearly 100% after full vaccination, and the protection period lasts 5–10 years (8). However, only 13.04% of medical institutions in Guizhou Province have the conditions for adult TTCV vaccination. Awareness of adult TTCV among medical staff is low, awareness of active vaccination among rural residents is generally insufficient, and the adult TTCV booster immunization strategy is not optimal, resulting in a lack of active immunization against tetanus (15). In addition, although all patients received passive immunotherapy after admission, they still failed to prevent the onset of the disease, revealing the limitations of passive immunity: the incubation period of this group of cases ranged from 1 to 27 days, while the protection time of equine tetanus immunoglobulin or tetanus antitoxin is generally only 10 days, and the longest protection time of tetanus human immunoglobulin is 28 days (generally 14–21 days), When the incubation period exceeded the protection time of passive immunization, the passive immunization in the body may have lost its effective concentration, resulting in the inability to reliably block the infection and pathogenesis of *Clostridium tetani* (16). Therefore, it is not reliable to rely solely on passive immunity. Active immunity is the primary means of preventing tetanus, and passive immunity can only be used as a remedial measure after exposure. In addition, only 21.74% of cases in this group received standardized wound treatment within 24 h of injury, and none of the non-survivors received it, suggesting that timely and thorough debridement, combined with passive and active immunity after injury, is key to preventing disease progression. It is recommended to conduct TTCV vaccinations before exposure among rural middle-aged and older farmers, improve access to standardized disposal within 24 h of injury, and accelerate the construction of tetanus prevention and disposal clinics at the grassroots level (8, 17).

The case fatality rate of tetanus in this study was 15.22%, lower than the globally reported 30–50% and higher than the 11% reported in the United States (18–20). However, all non-survivors occurred after family members decided to abandon further treatment, and none died during active rescue efforts. These cases of treatment abandonment were mostly middle-aged and older farmers from rural areas facing economic difficulties, who chose to interrupt treatment due to the high costs of treatment and uncertain outcomes in the later stages of the disease. Therefore, the observed case fatality rate in this study does not reflect the true natural history of tetanus; rather, it serves as an outcome indicator influenced by multiple factors, including disease severity, the family’s socioeconomic status, access to medical services, and the family’s perception of risk. It is essentially closer to an outcome under ‘restricted medical resources’ rather than a ‘mortality risk under standardized treatment conditions.’ This phenomenon indicates that access to treatment for severe tetanus in underdeveloped regions of western China depends not only on the level of medical technology but also on the patient’s family’s economic situation and the depth of coverage of the social medical security system. It is recommended to rely on medical insurance, medical assistance, and other systems to reduce the economic burden of intensive care for rural families and to avoid adverse outcomes from premature abandonment of treatment due to cost.

The results of univariate analysis show that the incubation period of the non-survival group was shorter than that of the survival group, and the severity classification of the non-survival group was severe and above, which was consistent with the conclusion that the short incubation period and the severity classification ≥ severe were the risk factors of tetanus death found by Chen et al. (21). The short incubation period typically indicates a larger quantity of tetanus bacilli invading the body, with rapid toxin production. The toxin can quickly and irreversibly bind to the nervous system, resulting in an acute onset and serious illness (16). At this time, even if a sufficient dose of tetanus passive immunotherapy is administered, it can only neutralize circulating toxin and is ineffective against toxins that bind to the nervous system. In this study, all non-survivors were associated with at least one complication, of which pulmonary infection, respiratory failure, hypoproteinemia, and multiple organ dysfunction syndrome were the most common. Studies have shown that tetanus spasm toxin can cause systemic skeletal muscle rigidity and laryngospasm, leading to aspiration and asphyxia, and then develop into aspiration pneumonia, hypoxemia, respiratory failure, and even multiple organ failure, which can lead to death in severe cases (1, 22, 23). It is suggested that airway management is the core link of severe treatment, which is consistent with the conclusions of Zhang and Espinosa (24, 25), that is, early preventive tracheotomy in patients with severe tetanus can effectively avoid airway obstruction caused by laryngospasm, thus significantly reducing the incidence of complications and mortality.

The median hospitalization cost for tetanus cases in this group was 55,724 yuan (equivalent to 8,359 US Dollars), with a maximum of 239,365 yuan (equivalent to 35,905 US Dollars). The median cost was equivalent to 3.51 times the per capita annual disposable income of rural residents in Guizhou Province in 2024. The economic burden was heavy, which increased the risk of rural patients ‘poverty caused by illness, and returning to poverty due to illness. Correlation analysis showed that hospitalization expenses were negatively correlated with the incubation period. The shorter the incubation period, the more severe the condition, the greater the intensity of monitoring and treatment required, and the higher the medical cost (8). Multiple regression analysis showed that hospitalization days and basic disease history were the influencing factors of hospitalization expenses. Hospitalization expenses increased with the number of hospitalization days, consistent with the study by Zhang et al. (26). The length of hospital stay is an important indicator of the efficiency of medical services and the economic burden of disease. Its extension directly leads to the consumption and cost accumulation of resources such as beds, nursing, and drugs. It is worth noting that the hospitalization expenses of patients with a history of underlying diseases in this study were lower. The possible causes were analyzed: the proportion of non-survivors among patients with underlying diseases was higher, and all non-survivors gave up treatment on their own, resulting in a higher proportion of patients giving up treatment on their own and relatively low hospitalization costs.

## 5 Limitations

There are some limitations in this study. First, only cases from some tertiary medical institutions in Guizhou Province are included; the sample size is small; and patients in primary hospitals and outpatient clinics are not covered, so there may be selection bias. Second, in this study, there were only 14 non-survivors, which is far below the required number of at least 10 outcome events per variable for the multivariable logistic regression analysis (event number/variable number ratio ≥ 10:1). Therefore, this study only conducted univariate comparative analyses to explore factors related to death and did not perform multivariable adjustments. The results should be considered exploratory and hypothesis-generating and need to be validated in larger sample sizes and multi-center studies. Third, all non-survivors in this study were due to family members giving up treatment, with none dying during the active resuscitation process. This selection bias may lead to an overestimation of the case fatality rate in this study, and caution is warranted when extrapolating to regions with universal insurance coverage or sufficient intensive care resources. Fourth, the failure to collect pre-hospital disposal costs, out-of-hospital treatment costs, and non-medical expenses may lead to an underestimation of the overall economic burden on patients. In the future, multi-center prospective studies should be conducted to expand sample size and include pre-hospital and indirect costs to more comprehensively assess the disease burden.

## 6 Conclusion

In summary, non-neonatal tetanus cases are mainly middle-aged and older male farmers, and most of them are not vaccinated with TTCV; the non-survivors were not treated within 24 h after injury, and the severity classification was severe and above, and all of them gave up treatment on their own. The case fatality and hospitalization expenses of this disease are high, and the disease burden is heavy. Hospitalization days are the main influencing factors of hospitalization expenses. It is recommended to strengthen health education on tetanus prevention among high-risk groups, such as farmers; promote pre-exposure TTCV vaccination and standardized post-exposure management; and reasonably control hospital stay while maintaining quality of care. Meanwhile, multilayer safeguards, including medical insurance and medical assistance, should be used to alleviate the economic burden on families with severe cases, prevent treatment abandonment due to cost, and ensure timely patient care. These measures can effectively reduce both the disease and economic burden on patients.

## Data Availability

The original contributions presented in the study are included in the article/supplementary material, further inquiries can be directed to the corresponding author.
